# The Disordered Cellular Multi-Tasker WIP and Its Protein–Protein Interactions: A Structural View

**DOI:** 10.3390/biom10071084

**Published:** 2020-07-21

**Authors:** Chana G. Sokolik, Nasrin Qassem, Jordan H. Chill

**Affiliations:** Department of Chemistry, Bar Ilan University, Ramat Gan 52900, Israel; chanasokolik@gmail.com (C.G.S.); nasrin_q86@hotmail.com (N.Q.)

**Keywords:** WASp interacting protein, protein–protein interactions, intrinsically disordered proteins, actin, cytoskeleton remodeling, SH3 domain, proline-rich motif

## Abstract

WASp-interacting protein (WIP), a regulator of actin cytoskeleton assembly and remodeling, is a cellular multi-tasker and a key member of a network of protein–protein interactions, with significant impact on health and disease. Here, we attempt to complement the well-established understanding of WIP function from cell biology studies, summarized in several reviews, with a structural description of WIP interactions, highlighting works that present a molecular view of WIP’s protein–protein interactions. This provides a deeper understanding of the mechanisms by which WIP mediates its biological functions. The fully disordered WIP also serves as an intriguing example of how intrinsically disordered proteins (IDPs) exert their function. WIP consists of consecutive small functional domains and motifs that interact with a host of cellular partners, with a striking preponderance of proline-rich motif capable of interactions with several well-recognized binding partners; indeed, over 30% of the WIP primary structure are proline residues. We focus on the binding motifs and binding interfaces of three important WIP segments, the actin-binding N-terminal domain, the central domain that binds SH3 domains of various interaction partners, and the WASp-binding C-terminal domain. Beyond the obvious importance of a more fundamental understanding of the biology of this central cellular player, this approach carries an immediate and highly beneficial effect on drug-design efforts targeting WIP and its binding partners. These factors make the value of such structural studies, challenging as they are, readily apparent.

## 1. Introduction

### 1.1. Scope

Modern biochemical research emphasizes the importance of complementing the biological and functional description of cellular events with a structural understanding of these on the molecular level. Such a combined structure-function view of biology—and the biomacromolecules that power it—has been repeatedly established as a prerequisite for studying biological pathways, analyzing signaling and regulation cascades, efficient drug design and optimization, and other investigation avenues that focus the majority of research efforts today. Fortunately, this state of affairs has motivated the development and advancement of experimental techniques capable of addressing this need, the main ones being X-ray crystallography, nuclear magnetic resonance (NMR), cryo-electron microscopy (cryo-EM), mass-spectrometry (MS), fluorescence-based spectroscopy, and a variety of scattering methods. All these bear the potential to provide a detailed mechanistic view of key cellular processes, as well as how they interface with each other. A case in point is WASp-interacting protein (WIP), a ubiquitous central participant in remodeling of the actin cytoskeleton, and therefore involved in regulation of activation and proliferation of cells [1,2]. While several excellent reviews have focused on the biology and protein-interaction networks of this cellular multi-tasker [3,4,5,6,7], less attention has been given to these interactions on the molecular level. To some extent, this is due to the disordered nature of WIP, which does not exhibit a stable three-dimensional structure, and adopts a more rigid conformation only upon interacting with its various binding partners. The recognized importance of intrinsically disordered proteins (IDPs) in biology is constantly increasing [8,9,10]. In this review, we hope to bring forth and summarize our structural knowledge of WIP and its main biological interactions. After a brief description of WIP and its relevant protein–protein interaction map, we will devote a section to each of the main interactions, highlighting structural information that has become known over years of research. Finally, we will attempt to outline prospects for future structural study of this important system.

### 1.2. WIP—Biology and Cellular Roles

WIP, a member of the verprolin family of actin-binding proteins [11], is a versatile and significant player in a number of biological processes. Originally discovered as a binding partner of WASp (Wiskott–Aldrich syndrome protein) [1], WIP has come into the spotlight in its own right. WIP shows highest expression in hematopoietic cells, and its most prominent function is regulation of the assembly of cytoskeletal actin filaments [3]. These form actin-rich structures, membrane protrusions and projections differing according to cell type in form and function, such as podosomes, filopodia, dorsal ruffles, stress fibers, lamellipodia and invadopodia, all involved in cell motility and migration, cell invasion through matrix degradation, cell adhesion, formation of synapses, or endo/exocytosis.

WIP’s function is crucial in immune cells, providing these cells with adhesive and migratory properties and an intact cortical actin cytoskeleton. WIP deficiency in B cells leads to distorted cortical actin and impaired signaling [12]. It is proposed that the actin cytoskeleton affects receptor diffusion and B cell surface receptor organization tuning receptor activation [12,13], which may be a mechanism relevant for other immune receptors as well [13]. Receptor-ligation in T cells and mast cells induces WIP-dependent actin polymerization and cytoskeletal rearrangement as a prerequisite for cell activation and proliferation [14,15,16]. WIP regulates the activation of both WASp, found in hematopoietic cells, and its ubiquitously expressed homolog N-WASp, nucleation-promoting factors (NPFs) that stimulate the molecular apparatus actin-related protein 2/3 (Arp2/3) complex to assemble filamentous actin. WIP also acts as a chaperone of WASp, protecting it from degradation and shuttling it to the sites of actin polymerization [15,17,18,19,20]. Research of the endocytosis mechanism in a yeast system suggests that threshold levels of WIP and WASp are needed to initiate actin assembly in the presence of a network of adaptor proteins, underscoring the central role of WIP and WASp in actin-nucleation scaffolds [21]. The pivotal role of the WIP-WASp complex in actin polymerization signaling is also exemplified by the fact that vaccinia virus and *Shigella* bacteria mimic regulators of the WIP-N-WASp complex (such as the adaptor Nck) to hijack the host’s actin machinery [2]. However, WIP has important WASp-independent functions as well, since cells containing WIP capable of binding WASp yet lacking the actin-binding domain showed decreased F-actin content and defects in T cell [22] and B cell [23] function, in agreement with the finding that the presence of WIP stabilizes F-actin and inhibits its depolymerization [3,18]. In addition, the central proline-rich domain of WIP serves as a scaffold for indispensable interactions with adaptor proteins, linking it to up-stream and down-stream regulators, as detailed in Section 3.2. Finally, WIP’s regulation of actin polymerization also affects maturation of neuronal cells and their synaptic activity [24].

WIP’s activity in promoting actin-rich structures also implicates it in many pathologies and makes its binding interfaces potential drug targets. Actin-rich membrane protrusions of cancer cells known as invadopodia degrade the extracellular matrix which allows cancer cells to migrate and form metastases, high WIP levels correlating with high invasiveness in breast cancer cells [25]. In addition, bacterial and viral pathogens, such as *Shigella flexneri* and Vaccinia virus, can recruit the host’s WIP-N-WASp complex to form actin-tails, propelling them and spreading infection [2,26]. As a regulatory protein, WIP impacts gene transcription and cell phenotype transitions. High WIP levels have been linked to enhanced stability of Yes associated protein (YAP) and transcriptional coactivator with PDZ-binding motif (TAZ) and oncogenic transformations [27,28,29]. WIP also controls through the G-actin/F-actin ratio the nuclear translocation of myocardin-related transcription factors (MRTFs), which in turn regulates the expression level of genes involved in focal adhesion as well as cancer cell migration and invasion [30]. Finally, the WIP-WASp complex affects T cell growth factor IL-2 gene transcription in T-cells through activation of the transcription factor NFAT which is needed for T cell proliferation [31].

### 1.3. Functional Domains and Sequences of WIP

**Figure 1:**
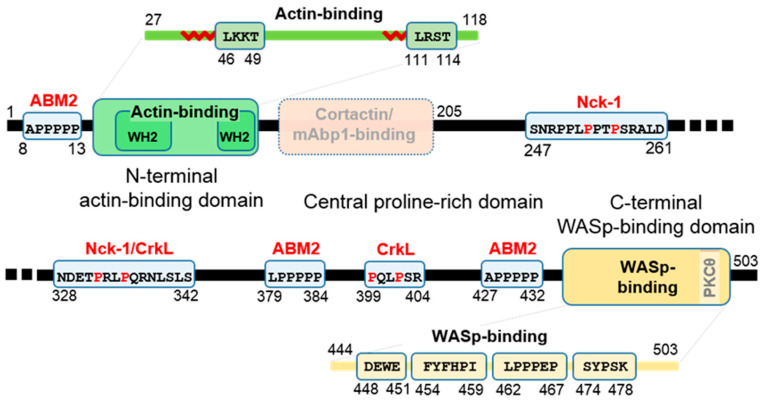
Functional domains of WIP. Schematic description of WIP (1–503) highlighting binding partners and motifs. Actin-binding, cortactin-binding, and WASp-binding regions are shown in green, pink (faded, indicating a putative binding domain), and orange, respectively. Polyproline motifs are shown in light blue (and extended in scale for clarity) with names of binding partners above (red). Sequence numbers are shown for motifs and domains. The actin-binding and WASp-binding regions are magnified (above and below, respectively) to highlight specific sequence features and epitopes. In the former, a red sawtooth pattern indicates the WH2 domain amphiphilic helix.

Figure 1 schematically describes functional sequences along the WIP polypeptide, with a major division into three regions, (i) the N-terminal actin-binding domain, (ii) the central proline-rich domain, and (iii) the C-terminal WASp-binding domain. The first (residues 1–120) is homologous to verprolin, a yeast protein involved in cytoskeletal organization, and includes two WASp homology 2 (WH2) domains (residues 32–59 and 96–118) [1] with G-actin binding sequences LKKT (residues 46–49) and LRST (111–114) separated by a highly flexible glycine-rich stretch. The second region (residues 121–440) contains proline-rich motifs that bind Src homology 3 (SH3) domains of NPFs, such as cortactin and its hematopoietic homologue HLCS1 and various adaptor proteins (details in Section 3.2.). In addition, the SH3 domain of the Src family tyrosine kinase Hck is known to interact with WIP directly in vitro, yet its binding motif/segment, assumed to be in region two has not been specified [32]. In addition, several SH3 domains of Pombe Cdc 15 homology (PCH) family proteins from T cells have been found to precipitate WIP through interaction with proline-rich motifs presumably in the second region [33]. The third (residues 441–503) binds to the Ena/VASP homology 1 (EVH1) domain of (N-)WASp [1,34] and contains a consensus protein kinase C θ (PKCθ) recognition site for phosphorylation on S488 [15]. The consensus motif for binding to profilin, an actin-regulating protein (xPPPPP, x = A/S/L/G), appears three times and is assumed to be an actin-based motility homology-2 (ABM-2) motif [3].

### 1.4. WIP Is a Disordered Polypeptide

WIP belongs to a class of proteins known as intrinsically disordered proteins (IDPs), defined as polypeptides lacking a well-defined secondary and tertiary structure under biologically native conditions [35,36,37]. This is a consequence of the WIP amino acid distribution, containing a low number (95 of 503, 19%) of hydrophobic residues and an excess of charged and polar residues (266 including Gly, 53%). In addition, WIP is rich in the disorder-promoting residue proline (142, 28%) that adopts locally rigid but globally flexible structures. As in other IDPs, the relatively small enthalpic gain of burying the few WIP hydrophobic residues that would normally drive the folding process is insufficient to compensate for the concomitant loss of entropy [38]. Although this lack of structure contradicts the structure-function paradigm that has motivated decades of protein investigations, IDPs have recently re-ignited the interest of the structural biology community as the idea of function without structure gains acceptance. It is now undisputed that IDPs are intimately involved in all central cellular processes, including gene expression, cell-cycle control and malignancy, signal transduction, protein aggregation and degradation, and are also disproportionally involved in human disease [39,40,41].

As is clear from the previous sections, WIP could be considered archetypical of this class of proteins. Characteristically, WIP can be described as an array of short interaction domains, each possessing independent binding capabilities, beaded together on a ‘necklace’ formed by connecting non-functional segments. However, contrary to multi-domain structured proteins, each of these ‘beads’ is actually an ensemble of rapidly interchanging unfolded and partially-folded states which on aggregate account for overall behavior in solution [42,43]. Accordingly, the energetic conformational landscape of such domains is a multi-minima surface lacking a distinct low-energy state. This description is consistent with the role of WIP as a multi-tasking interaction hub, with an ability to recruit proteins and elicit specific functionalities. As will be demonstrated below, it is clear that unstructured domains of WIP are induced to fold to specific structures upon binding of interaction partners. Typically for an IDP, the unfolded state of WIP is conducive to post-translational modifications (PTMs) [44], the main one being phosphorylation, and the coupling of multiple protein–protein interactions with their PTM-based modulation results in the potent regulatory network for which WIP is well-known. With the importance of IDPs on the rise in recent years, structural methods have evolved to address this intriguing class of proteins [45,46,47,48,49,50,51,52].

Beyond the phenomenological observation of the biological importance of IDPs, there remains the mechanistic question of how they exert their biological function in the absence of structure. As do the majority of IDPs, the encounter between a globular interaction partner and an unstructured functional domain of WIP induces the folding of the latter into a specific structure, with the binding protein serving as an ‘external’ hydrophobic core. Given that the typical WIP interaction domain is actually an ensemble of conformations, the binding protein could ‘select’ an appropriately quasi-folded conformation, or conversely induce a compaction of an unstructured conformation upon contact between binding surfaces. Determining the relative contributions of these two mechanisms is a fundamental question of IDP biology [53,54]. Hybrid mechanisms, in which residual disorder exists even in contact with the binding partner, have been described in some IDPs and are known as ‘fuzzy complexes’ [55,56,57]. Not surprisingly, the entropic penalty of a collapse of several possible states of WIP into a single bound state leads to complexes of varying affinity levels, and dissociation constants in the 0.1–100 μM range are known. This also highlights the challenging nature of WIP structural biology, since weak complexes often defy structural study by static approaches (crystallography, cryo-EM) and require methods that preserve molecular dynamics such as NMR, fluorescence, or scattering-based methods.

### 1.5. Rationale and Structure of Review—List of WIP Interaction Domains

**Figure 2:**
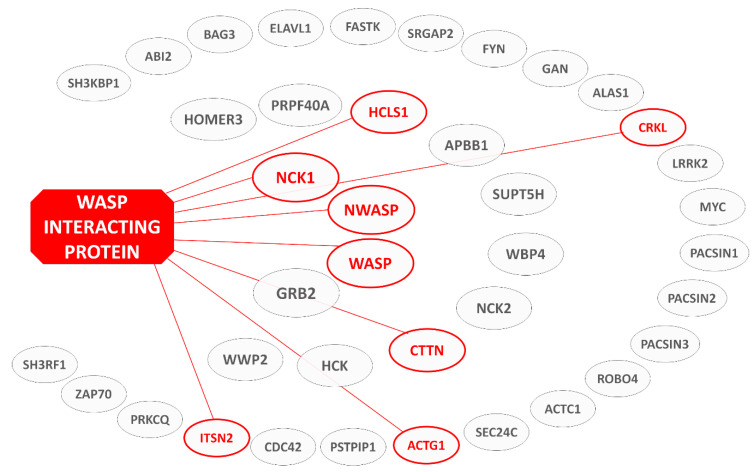
Interaction partners of WIP. The Human Integrated Protein-Protein Interaction rEference (HIPPIE) database [58] indicates proteins with a good probability (*p* ≥ 0.5) of interacting with WIP. Full protein names can be found in Table S1. Inner circle—0.94 ≤ *p* ≤ 0.99, middle circle—0.68 ≤ *p* ≤ 0.86, outer circle—0.52 ≤ *p* ≤ 0.63. WIP binding partners mentioned in the structural context of this review are highlighted in red.

The previous sections emphasize the central role played by WIP in a variety of cellular processes, and as a consequence the importance of understanding the molecular mechanisms underlying its interactions with its multiple binding partners and activity. In light of this, and the aforementioned relative paucity of such data, herein we aim to curate the available structural information on the cellular interactions of WIP. To place this molecular-level view in its biological context, Figure 2 illustrates the wingspan of WIP in terms of the proteins it engages in various stages of cellular homeostasis and highlights interactions for which structural information is available. This serves as a graphic illustration of the ‘interaction hub’ role assumed by WIP, while emphasizing sobering gaps of information (to be addressed by future investigations) separating the few interaction systems that have been well characterized. By nature, these interactions will form the focus of the following sections.

## 2. The Actin-Binding Region

### 2.1. Actin—A Cytoskeleton Protein—and Actin-Binding Domains

**Figure 3:**
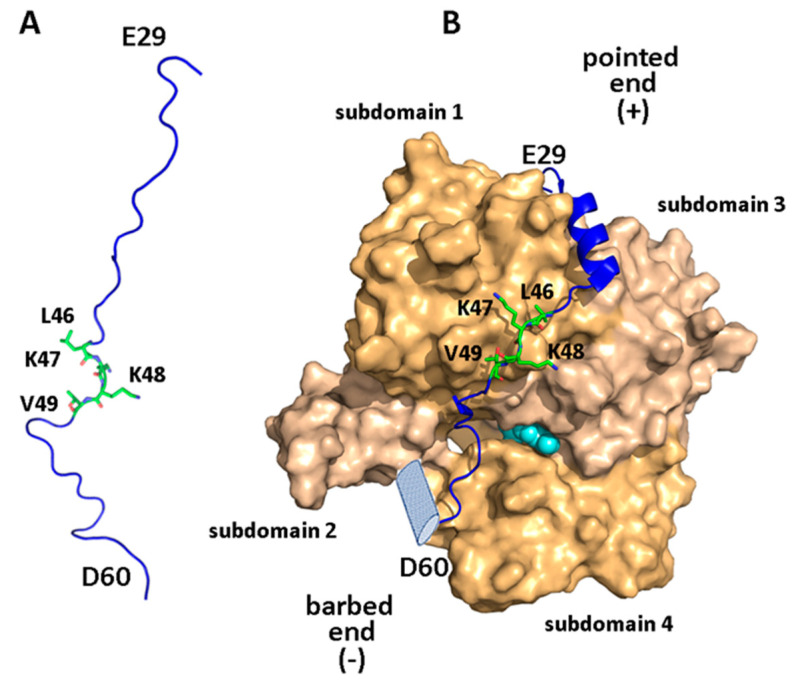
Structural view of the WIP-N/actin interaction. (**A**) A plausible representation of residues 28–61 of WIP (blue) and sidechains of residues 46–49 in their free form, (**B**) bound WIP(28–61) in complex with actin (pale/dark orange, PDB ID: 2A41 [68]), showing actin subdomains. Residues of the actin-binding LKKV motif are highlighted in green. The putative C-terminal helix (absent in WIP but present in other actin-binders) is portrayed as a light-blue cylinder, and the bound nucleotide (between subdomains 3 and 4) is shown as cyan-colored spheres.

Actin is a cytoskeletal protein found in most eukaryotic cells. It participates in many crucial cellular processes, including muscle contraction, cell motility and migration, division and signaling, immune surveillance, angiogenesis, tissue repair, phagocytosis, and cell regeneration [59,60]. The constant and rapid reorganization of the actin microfilament system accompanying these depends on nucleation, elongation and depolymerization of actin filaments, and therefore cellular reorganization of actin is highly regulated [60]. Actin exists in two different forms in equilibrium, monomeric (globular, or G-) and polymeric (filamentous, or F-) form. The dynamic equilibrium between G- and F-actin is central to cellular behavior and is regulated by extracellular stimulation [61]. Monomeric G-actin, the basic unit for actin filaments, contains four subdomains: subdomains 1 (residues 1–32, 70–144, 338–374) and 2 (33–69) of the small main domain, and subdomains 3 (145–180, 270–337) and 4 (181–269) of the large main domain [61,62]. As shown in Figure 3, together these four subdomains create two structural clefts, a large nucleotide-binding cleft between subdomains 2 and 4 and a hydrophobic target-binding groove between subdomains 1 and 3 [62,63]. The former is the center of the enzymatic catalysis site where hydrolysis of ATP and binding of divalent cations (Mg^2+^ or Ca^2+^) takes place, mediated by residues 11–18 and 154 [61]. The latter modulates the binding affinities of actin-binding modules (ABMs), leading to changes in the stability of the actin filament.

ABMs are actin-binding entities that control the formation of the actin cytoskeleton by regulating the transition between G- and F-actin in cells [64,65]. G-actin bound to ABMs or proteins of the profilin family is the major source of actin monomers for filament nucleation and elongation [66], and other roles of ABMs include disengaging, capping, and monomer sequestration. ABMs share a conserved motif that competes with actin for a common binding site. A main contributor to this essential site is a hydrophobic pocket that mediates significant interaction of actin complexes [62]. The hallmark of ABMs is a 9–10 residue segment that upon binding to the barbed end of actin forms a helical region followed by a conserved LKK(T/V) motif (with some variations). The five residues that follow this sequence play a key role in determining how the extended chain interacts with actin. Generally accepted is the subdivision of ABMs into WH2 domains, characterized by longer conserved regions preceding the amphiphilic helix, and β-thymosins, identified by a conserved linker connecting the helix and the LKK(T/V) motif and a second C-terminal helix following these that interacts with the pointed face of actin [67].

### 2.2. Structural Aspects of the WIP-Actin Interaction

WIP and its homologs CR6 and WICH/WICH contain N-terminal ABMs belonging to the WH2 family [59,62,68]. In WIP, these span residues 32–60 (a ‘long’ WH2 domain) and 96–118 (a ‘short’ WH2 domain), including the conserved sequences L^46^KKT^49^ and L^111^RST^114^, respectively [11]. The crystal structure of the first of these ABMs in complex with actin (PDB ID: 2A41 [68]) revealed the structural details of this interaction. Residues 33–42 form a three-turn amphiphilic helix that embeds its hydrophobic face, including residues L36, L37, and I40, in a cleft at the barbed end of actin, and basic residues K47/K48 of the conserved motif are positioned close to a negatively charged surface including actin residues D24/D25, E99/E100 (subdomain 1), and E334 (subdomain 3). Characteristically for ‘long’ WH2 domains, the following segment (residues 52–55) runs parallel to the actin subdomain 1 β-sheet [69]. This creates an extensive binding interface (absent in ‘short’ WH2 domains) that includes a salt bridge between R54 and actin residue E93, while the small side-chain of adjacent S55 allows deeper penetration into the nucleotide cleft of actin [62,68,70,71]. The affinity to actin of smaller WIP fragments consisting of residues 29–46 and 46–63 drops 10-fold and over 1000-fold, respectively, demonstrating the importance of the amphiphilic helix in binding actin [68].

Since only a minor fraction of cellular WIP is in the actin-bound state, the ensemble of conformations adopted by its ABM sequences in the intrinsically disordered free form is relevant to their cellular behavior. NMR-based measurements were employed to characterize the conformational ensembles of residues 2–65 of WIP containing the N-terminal ABM. Secondary backbone chemical shifts, temperature-induced chemical shift effects, backbone heteronuclear coupling constants, and analysis of residual dipolar couplings for this segment all concurred in identifying a helical propensity for residues 30–42 and partial extended β-strand character for residues 45–62. These propensities echo the ABM actin-bound structure, suggesting this pre-formed conformation may contribute to the actin binding mode [72,73]. As shown by changes in backbone J-couplings, this structural bias in the WIP conformational ensemble was obviated by exposure to denaturing conditions [73]. Interestingly, a lysate mimicking actin-deficient cellular crowding effects found a decrease in these structural tendencies, presumably due to non-specific protein–protein interactions offering higher stabilization to unfolded conformations of the ABM. Thus, in the cellular environment, the ABM may be less structured than in its purified form. Notably, a partially pre-formed β-strand similar in significance to residues 45–62 was observed connecting the profilin-binding and amphipathic helix sequences (residues 17–25), a region highly conserved in WIP and its homologs. This may indicate a potential role for this linker in mediating the binding of actin, possibly by interacting with a yet-unknown binding partner [73].

## 3. The Proline-Rich Intermediate Region

In vitro and in vivo biological studies have pinpointed the proline-rich domain as a frontier of high clinical relevance with interaction motifs that are either heavily implicated in cancer metastasis formation [74,75] or may be vital for proper immune system functioning [15,76]. Thus, it is surprising to find that this major WIP segment has not been structurally investigated, particularly when compared to the terminal domains described in other sections of this review. Possibly because SH3/polyproline complexes have been characterized back in the 1990s, they have been considered research targets with less potential of novelty. However, this may be a misconception, as many issues of binding specificity, molecular determinants of affinity, and effects of extended motifs are extremely important for inhibitor design and remain largely unresolved. Another potential barrier faced by structural studies is the moderate affinity of these interactions that hinder both crystallization efforts and solution NMR investigations. Indeed, there is a lack of biophysical characterizations of these complexes using methods such as isothermal calorimetry (ITC) and microscale thermoephoresis (MST) for quantification of affinity, NMR, X-ray crystallography, and small-angle X-ray scattering (SAXS) for structure determination of the complexes, or NMR and single-molecule fluorescence techniques to characterize their dynamic nature. We therefore focus on more qualitative biological studies of these interactions.

### 3.1. SH3 Domains and Their Ligands

**Figure 4:**
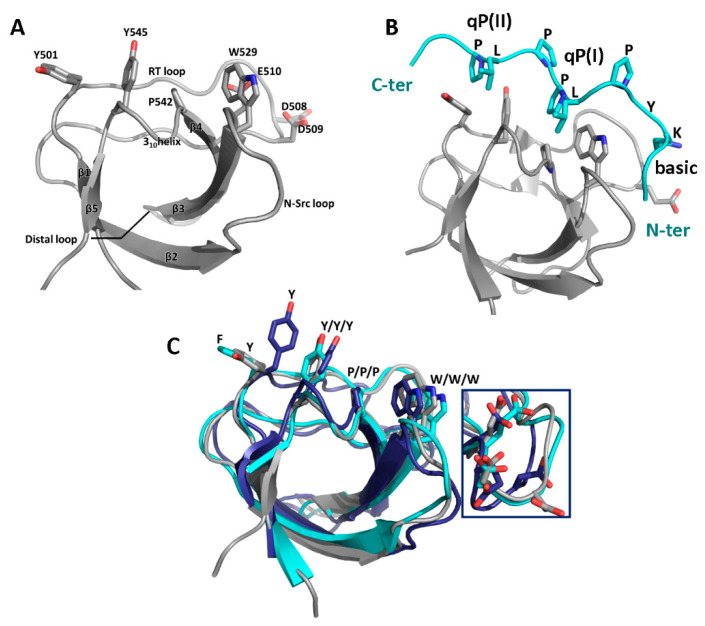
Common structural features of WIP-binding SH3 domains. (**A**) The cortactin SH3 domain showing the characteristic features with numbering according to human cortactin (PDB ID: 5NVJ) [87]. (**B**) Complex of a Hck SH3 domain (PDB ID: 2OJ2) [88] with a high-affinity class I peptide ligand KYPLPPLP showing the typical ligand PPII conformation and placement in binding grooves: The two LP dipeptides interact with the aromatic residues, while the N-terminal lysine of the ligand interacts with glutamate of the specificity pocket. (**C**) Overlay of the following SH3 domains, cortactin (grey, PDB ID: 5NVJ [87]), Nck SH3.2 (cyan, PDB ID: 2JS0 [89]), and N-terminal CrkL domain (blue, PDB ID: 2LQN [90]), demonstrating the high similarity of all SH3 domain structures. Key residues of the hydrophobic binding grooves are shown in stick representation. Inset shows an overlay of specificity pocket acidic residues that form salt bridges with the ligand.

Src Homology-3 (SH3) domains are small modules of protein–protein interactions found in signaling and regulatory proteins. They usually are composed of 55–70 residues [77] with 5–8 β-strands arranged as two anti-parallel β-sheets or a β-barrel, with three loop regions, the RT loop (between β_1_-β_2_), N-Src loop (β_2_-β_3_), and distal loop (β_3_-β_4_), and a short 3_10_-helix (β_4-_β_5_) [78]. Two of the three ligand-binding grooves are formed by highly conserved (mostly) aromatic residues, including a tryptophan (often the first in a β_3_-WW motif), two additional aromatic residues (tyrosine or phenylalanine) located in the RT-loop and the 3_10_ helix, and a proline residue at the end of β_4_ (see Figure 4A) [79]. Their sidechains adopt an orientation essentially unchanged by ligand binding, suggesting a preformed template [77].

SH3 ligands are proline-rich motifs of disordered protein segments that form a left-handed polyproline (PPII) helix seen as arches that place the (i) and (i + 3) residues at the same height (Figure 4B), usually with a qPxqP sequence. One qP dipeptide binds to each hydrophobic groove, q being a hydrophobic residue [78,80]. Ligand motifs include flanking basic residues which interact with acidic RT-loop residues in a third pocket called the canonical specificity pocket (the acidic residues seen behind tryptophan and marked in Figure 4, A and B and detail in C) [79]. Consensus ligands are classified as class 1 (consensus motif RxLPPxP) or class 2 (xPPLPxR), characterized by basic residues at the N- and C-terminal side of the PxxP motif, respectively and bind in opposite orientations [78]. Although all SH3 domain structures are highly similar and consensus motifs show small variations, SH3 domains do recognize specific ligands and, conversely, ligands recognize specific SH3 domains, to a certain degree. In particular, ligand interactions with RT-loop, N-Src loop, and β_4_ residues have been implicated in mediating both affinity and specificity. On the ligand side, residues outside the core binding motif have been associated with affinity and specificity [79,81,82,83,84,85]. In addition, non-canonical binding with recognition of non-PxxP ligands is not uncommon for certain SH3 domains and its prevalence may be underestimated [78,79,82,85,86].

### 3.2. Binding Partners and Binding Motifs

As mentioned above, information on WIP binding partners is often limited to identification of the interacting protein and, in some cases, the interacting segment or binding epitope sequence. This was generally obtained using biological methods, including yeast-two-hybrid assays, pull-down assays with immobilized SH3 domains followed by Western blot analysis of the binding partners, and further pull-down assays using purified WIP to verify direct binding. Deletion mutants were then used to identify WIP binding segments and/or assess the various affinities in cases of multiple SH3 domains. Alternatively, mutations of the critical SH3 tryptophan residue to lysine resulting in loss of affinity were employed to confirm SH3-mediated binding and ligand-SH3 domain pairings. Techniques used in cells were co-immunoprecipitation, fluorescence assays to verify co-localization and reveal cellular distribution, and assays to assess the cellular effects of binding. Table 1 lists the interaction partners discovered through these techniques.

Very rudimentary information is available for mammalian actin-binding protein 1 (mAbp1) and cortactin, two proteins with a similar domain organization including an N-terminal F-actin binding motif and a C-terminal SH3 domain. The high sequence identity of their SH3 domains (62% amino acid identity of mAbp1 and cortactin) suggests interaction with the same ligands [91]. A yeast-two-hybrid assay identified WIP residues 136–205 as a cortactin-binding segment while cortactin failed to interact with full-length WIP lacking residues 110–170 (Δ110–170) [92]. mAbp1, too, was found to bind WIP, and deletion of WIP residues 110–170 reduced the interaction by more than 70% [93]. The W→K mutation of the binding site tryptophan of both the cortactin and mAbp1 SH3 domains, W525K and W415K respectively, blocked binding of cortactin/mAbp1 to WIP, confirming SH3-mediated binding for cortactin and mAbp1 [92,93]. In addition, binding of WIP to the hematopoietic homologue of cortactin, hematopoietic lineage cell-specific protein 1 (HLCS1) was proven and W→Y mutation of the HLCS1 SH3 domain abolished binding [94]. Notably, the dissociation constant for the complex of full-length cortactin and WIP was estimated as 0.3 µM by densitometry (based on the correlation between the concentration of a complex and the intensity of a Western-visualized SDS-PAGE band), constituting a relatively high affinity for SH3-mediated interactions [92]. Similarly, the intersectin adaptor proteins intersectin-1 (ITSN1, the short variant ITSN1-S and the long variant ITSN1-L) and intersectin-2 (ITSN2) have been found to interact with the 318–450 and 13–450 segments of WIP respectively (overlapping the CrkL/Nck sites, see below), both omitting the N-WASp-binding segment to confirm that the interaction is not mediated by N-WASp [95], while in yeast-two-hybrid assays the 353–503 segment interacted with ITSN2, among others [96]. In vitro binding assays indicated that of their five different SH3 domains (labeled A–E), the interaction with WIP occurs via the A/C/E domains, whereas the B/D domains have no WIP affinity [95].

Specifically located binding motifs have been suggested for only two WIP binding partners. Yeast-two-hybrid assays mapped the Crk-like protein (CrkL) binding site in WIP to the 321–415 region, and established that WIP residues 321–376 and 377–503, but not 416–503, interact with CrkL, suggesting two binding sites in residues 321–376 and 377–415. An additional yeast-two-hybrid assay mapped the CrkL WIP binding site to the N-terminal SH3 domain (SH3.1), while the SH3.2 domain failed to interact with WIP. The 321–415 segment contains two copies of the Crk SH3.1 consensus binding motif PxLPx(K/R) [97], in residues 332–337 and 399–404, in complete agreement with the yeast-two-hybrid assay [15].

The most detailed information is available for the WIP–Nck interaction. The adaptor Nck is composed of three tandem SH3 domains (SH3.1, SH3.2, and SH3.3) followed by one SH2 domain. The latter interacts with phosphotyrosine residues in ligand-activated receptor tyrosine kinases (RTKs) and transmits the signals to effector molecules (such as WIP) interacting with its SH3 domains [98]. Affinity-precipitation of WIP with individual Nck SH3 domains demonstrated that WIP bound to SH3.2, but poorly to SH3.1 and SH3.3. Mapping of the Nck-binding site of WIP by yeast-two-hybrid system demonstrated binding to a region spanning residues 321–415 [98]. A peptide-array analysis consisting of WIP-derived 15-residue segments revealed that Nck-binding is mediated by class 2 peptide sequences SNRPPLPPTPSRALD (residues 247–261) and NDETPRLPQRNLSLS (residues 328–342), both sharing the PxxPxRxL motif, while the second one conforms also to the Nck SH3.2 consensus motif PxxPxRxxS [99]. Alanine substitution of the proline residues in the PxxPxR motif abolished Nck binding by the peptides in vitro. Alanine substitution in both motifs was needed in WIP mutants to eliminate Nck binding completely, indicating that each motif can bind Nck independently. Selective affinity of these peptides to SH3.2 was confirmed upon observing that the W143K mutation (in SH3.2), but not W38K (SH3.1) or W229K (SH3.3), was sufficient for eliminating Nck binding [26]. A summary of SH3-binding WIP epitopes appears in Table 1.

## 4. The WIP-C/WASp Interface

### 4.1. Structure and Binding Epitopes in the WIP-C/N-WASp Complex

**Figure 5:**
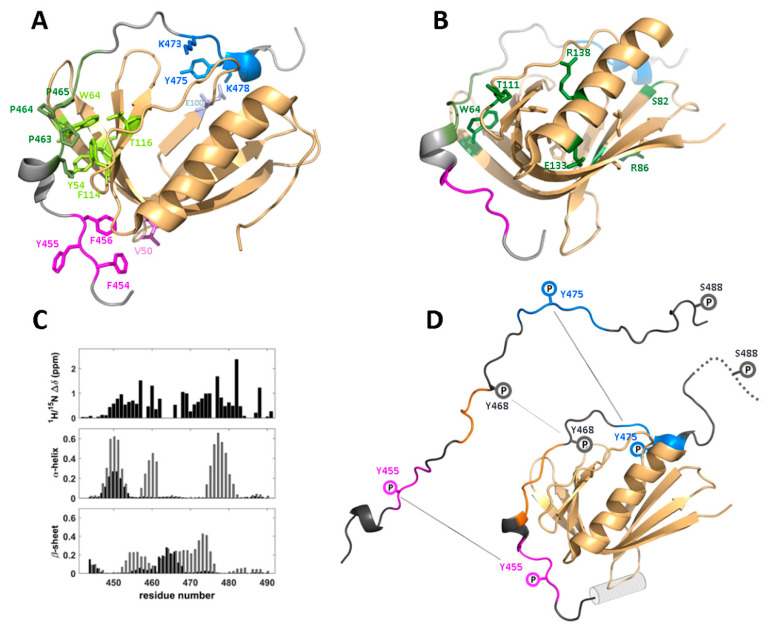
Structural view of the interaction between the C-terminal domain of WIP and the WASp EVH1 domain. Structures are based on the complex between WIP residues 451–485 tethered to residues 26–147 of rat N-WASp (PDB ID: 2IFS [104]). Residue numbers are based on the analogous WASp sequence. (**A**) Structure of the WIP-N-WASp complex (adapted from [104]). N-WASp is shown in light orange, and three WIP epitopes are shown in magenta, green, and blue. Sidechain atoms of these epitopes and key N-WASp residues forming the binding interface are shown as sticks with a similar coloring scheme. (**B**) Distribution of WAS-causing mutations; residues that when mutated result in severe WAS, are highlighted with sidechains in stick representation. Buried mutation hotspot residues are colored in light-orange, and surface-exposed hotspot residues are colored in green and labeled. T111 represents the location of analogous N-WASp residue R601. (**C**) Chemical shift data indicating a binding-induced conformational change in WIP, including (top) HSQC perturbations along the sequence, (middle) predicted helical content for free (black) and bound (gray) WIP, (bottom) same as previous but for β-strand content. (**D**) Model of binding induced changes in residues 442–492 of WIP showing the additional helical motif binding to WASp (gray cylinder) and phospho-sites along the sequence.

The interaction—for which WIP is named—between the C-terminal domain (last 50–60 residues) of WIP and the N-terminal EVH1 domain of WASp/N-WASp has been well characterized both biochemically and structurally. WIP was first identified by a yeast two-hybrid assay that linked it to WASp [1], and the interaction was pinpointed a few years later to the WASp EVH1 domain [2], consistent with the location of several WAS-causing mutations in this region [15,101,102,103]. NMR-based structure determination of complexes between short (residues 461–485) and extended (451–485) WIP-derived peptides fused to the EVH1 domain of N-WASp, an ubiquitously expressed homolog of WASp, revealed the molecular basis of this interaction in detail (Figure 5A) [34,104]. The most striking feature of the complex is the extensive interface involving multiple epitopes along the WIP sequence. Specifically, the canonical EVH1-binding polyproline motif (DLPPPEP, 461–467) nestles into a groove on the second EVH1 β-sheet formed by the characteristic tryptophan residue W64 and conserved residues Y54, F114, and T116 (all numbering based on the WASp sequence). However, this buried surface is flanked by two additional interaction regions, a hydrophobic motif (FYFHPIS, 454–460) identified in an earlier pull-down assay [105] interacting with ‘bend’ residues V50/V51 of the β-sandwich, and a helical motif (KSYPSK, 473–478) forming a salt bridge between K478 and residue E100 [34,104].

In its commonly found WASp-bound state, WIP adopts a tightly constrained conformation that positions the binding epitopes near their respective EVH1 interaction surfaces. Some secondary structural elements induced by EVH1-binding are also transiently present in free WIP, although this region is intrinsically disordered so that there is no prevalent conformation. An analysis of secondary chemical shifts and solvent exchange protection factors along the backbone of a C-terminal (residues 407–503) WIP domain revealed a structural propensity echoing the structure of EVH1-bound WIP residues 461–485, a lefthanded polyproline helix followed by a helical segment for residues 462–467, and 474–478, respectively. Thus, the complex may form by a conformational selectivity mechanism, accompanied by a tightening of the flexible linker (residues 469–472) between these two motifs. More importantly, the analysis also identified a previously undetected fourth segment (residues EDEWES, 447–452) with a strong helical tendency and high conservation level (DDFE, residues 417–420 in CR16, or 394–397 of WICH), suggesting a potential involvement in EVH1 binding (Figure 5C,D) [106]. Indeed, an NMR investigation of a complex of the T cell WASp EVH1 domain bound to an extended WIP polypeptide including this additional epitope (residues 442–492) showed the DEWE segment to adopt a turn conformation and interact with a helical segment (ENQRLFE, WASp residues 31–37) preceding the β-sandwich and overlooked in earlier structural studies [107]. Homologs of this additional helix appear in N-WASp (ENESLFT, residues 23–29) and in related pleckstrin homology (PH) domains [108,109], suggesting it should be included in the functional EVH1 domain.

### 4.2. Functional Implications of the WIP/WASp Interface

Over half of WAS-inducing mutations are missense mutants in the EVH1 domain (http://www.hgmd.cf.ac.uk, search term WAS, and [34]), emphasizing the importance of the WIP/WASp interaction. Structural data for the N-WASp/WIP and WASp/WIP complexes illuminate the mechanism by which these mutations exert their deleterious effect. Of 19 mutations identified as causing strong/severe WAS, ten (L35, C73, F74, V75, W97, H115, G125, L126, F128, A134, numbering based on the WASp sequence) are buried amino acids (defined as *f*_ASA_ < 0.2, where *f*_ASA_ is the side-chain fractional accessible solvent area), and likely to cause the disease by disrupting native WASp structure. Specifically, W97, H115, F128, A134 form a packing unit (together with Y107 that is hydrogen-bonded to H115) that directly affects the polyproline-binding groove, and other residues of this group are located in the hydrophobic core of the β-sandwich structure. The other surface-exposed nine mutations (S24, E31, L39, W64, S82, R86, T111, E133, R138) may directly impact WIP binding, as in the case of conserved polyproline-interacting residue W64, but also indirectly, as in the case of the helix-destabilizing mutation R138P that may affect the β-sandwich structure (Figure 5B) [110]. Particularly interesting is mutation hotspot R86, for which four different mutation phenotypes are known, and confirmed by yeast two-hybrid assay [103], located on the WASp face diametrically opposed to the polyproline binding site. Contrary to a previous hypothesis, the NMR analysis did not suggest a direct contact with WIP in this region, and it is also possible that its interaction with nearby negatively charged residues, including the critical E100 (homologous to N-WASp E90 forming an intermolecular salt-bridge) is the WAS-causing factor.

In cell imaging FRET techniques used to identify WASp/WIP dissociation in cells expressing various WIP mutants provided further insight into the positioning and contribution to WASp/WIP function of various WIP epitopes. While WIP mutated at the FYF (454–456) epitope lost the ability to bind WASp, loss of the polyproline and DEWE (448–451) epitopes incurred equally significant reductions in affinity to WASp. In addition, of all epitopes, the DEWE was shown to have the greatest effect upon ubiquitylation levels, indicating that this additional binding interface was important for protecting WASp from proteasomal degradation [107]. The mechanism by which this occurs in yet unclear, since confirmed WASp ubiquitylation sites K76 and K81 [111] are distant from DEWE interaction surface, and it is possible that this interaction interferes with another component of the ubiquitylation machinery.

### 4.3. Phosphorylation-Induced Dissociation of the WIP/WASp Complex

It is well established that phosphorylation-induced dissociation of the WIP C-terminal domain from WASp mediates both activation and eventual proteasomal degradation of WASp, but the molecular mechanism underlying this phosphorylation has been controversial. Soon after identification of the WIP/WASp interaction, it was shown that PKCθ-mediated phosphorylation occurring on S488 (in the sequence RSGSNR, residues 485–490) is correlated with dissociation in Jurkat cells, and that the S488D phospho-mimicking mutation in WIP abolished its affinity to WASp [15]. In contrast, a later study showed similar levels of WASp pulldown by WIP and its unphosphorylated (S488A) and phosphorylated (S488D) mimicking mutants [31]. However, a more recent in-cell molecular imaging approach attributed this to an independent actin-mediated interaction surface between the two proteins, and by following the movement of WIP-containing clusters in real time established a clear difference between unphosphorylated and phosphorylated mutants and directly implicated PKCθ phosphorylation at S488 as a mediator of complex dissociation [112]. Surprisingly, this proposed mechanism has found little structural support. Structures of single-chain tethered complexes (in which the WIP polypeptide was connected to the N-WASp N-terminal) were either missing the relevant residues [34,104] or unable to observe changes induced by phospho-mimicking mutants S488D/S488E [113]. The later NMR-based analysis of the WIP/WASp complex did not find secondary structure differences between free and bound WIP for residues 483–492, and WIP dissociation-inducing mutations (as shown in cells) caused little (if any) change in chemical shifts within this region [107]. Concomitantly, introduction of the phospho-mimicking S488E mutation had no effect on WASp resonance frequencies (Halle-Bikovski A, Baluom S, Chill JH, unpublished results). The lack of structural evidence from these systems supporting PKCθ-mediated dissociation hints at a possible indirect effect on the state of the WIP/WASp complex. Phosphorylation on tyrosine residues in the WASp-binding domain, specifically Y455, Y468, and Y475 by Bruton’s tyrosine kinase (BtK) has been suggested as an alternative inducer of dissociation [114]. This appears to be in better agreement with available structural information, since these phospho-sites reside well within the proven WIP interaction surface (Figure 5D), but further studies would be required to provide experimental support of this notion.

## 5. Discussion and Summary

WIP is a multi-tasking protein forming a ‘hub’ of protein–protein interactions, and is involved in a variety of inter-connected and intricately regulated biochemical pathways. Although its first discovered role was in mediating the immune response, much research since has established important functions in cytoskeletal changes via its interaction with G- and F-actin under different conditions, regulation via interaction with several adaptor proteins, and maturation and synaptic activity of neuronal cells. Commensurately to its wide-ranging biological roles, WIP is involved in several pathological conditions and has become recognized as an important biomarker of aggressive cancer [28,74]. Many hypothesized WIP epitopes have been identified using only bioinformatics methods and lack experimental verification. Even so, important binding epitopes within the three major WIP sections have been investigated structurally, either by full structure determination or by structural biophysical approaches. In some cases, the average conformation adopted by such epitopes in their free form exhibits a structural propensity that is reminiscent of the epitope in the bound structure, hinting at a plausible conformational selectivity mechanism of binding. However, this effect may be sequence dependent. Some epitopes will be more ‘pre-formed’ due to local conformational constraints (i.e., in the case of a polyproline motif), while other epitopes may adopt a conformation corresponding to an alternative local energetic minimum, only to be ‘re-configured’ upon interaction with a binding partner.

These important structural studies have invariably required a reductionist approach, in which each interaction epitope in complex with its binding partner is treated independently. The simplification achieved in such studies must constantly be weighed against the potential loss of biological context, specifically the interdependence of such interaction pairs and/or the possibility of multi-protein interactions. This highlights the importance of complementary cellular and in vivo studies, based on fluorescence cellular imaging or in-cell NMR, in addressing this concern by offering a more holistic and potentially temporally resolved view of the network of WIP interactions that is central to its biological function. Ultimately, a multidisciplinary approach to WIP structure-function studies (and other disordered proteins), in which critical epitopes are first predicted and later investigated by qualitative and quantitative structural approaches, is a promising path towards a better understanding of key biological processes on the molecular level, with potential therapeutic implications.

## Figures and Tables

**Table 1:** Summary of SH3-binding WIP epitopes.

Partner	WIP Segment	Binding Motif	Effect	Ref.
Cortactin SH3 (NPF)	136–205	Not determined (ND)	Increases cortactin’s activation of the Arp2/3 complex, cortactin recruits WIP in invadopodium formation	[75,92]
mAbp1 SH3 (adaptor)	110–170	ND	Regulates dorsal ruffle formation	[93]
ITSN1-S/ ITSN1-L 1st/3rd/5th of 5 SH3 domains (adaptor)	318–450	ND	enhances association of ITSN1 with N-WASp and β-actin, facilitates formation of filopodia-like protrusions, regulates intra-cellular vesicle trafficking	[95,100]
ITSN2 1st/3rd/5th of 5 (adaptor)	13–450	ND		[95,96]
CrkL 1st SH3 of 2 (adaptor)	321–415	^332^PRLPQR^337^ (class 2) ^399^PQLPSR^404^ (class 2) (comply with Crk SH3.1 consensus binding motif PxLPxK/R)	Presumably preformed CrkL-WIP-WASp complex associates with phos-ZAP70 after T cell receptor (TCR) ligation	[15]
Nck-1 2nd SH3 of 3 (adaptor)	247–261 328–342	^247^SNRPPLPPTPSRALD^261^ ^328^NDETPRLPQRNLSLS^342^ (both class 2, 328–342 complies with the consensus motif for Nck SH3.2 PxxPxRxxS)	Couples extracellular signals to cytoskeleton assembly system	[26,98,99]

## Supplementary Materials

The following are available online at https://www.mdpi.com/2218-273X/10/7/1084/s1, Table S1: WASp interacting protein binding partners from HIPPIE database.
