# Supplementary material for: The Disordered Cellular Multi-Tasker WIP and Its Protein–Protein Interactions: A Structural View
Source: Biomolecules. 2020 Jul 21;10(7):1084. doi: 10.3390/biom10071084 (PMC7407642; doi:10.3390/biom10071084)
Supplement: Supplementary file 1 [file biomolecules-10-01084-s001.pdf]

Table S1: WASp interacting protein binding partners from HIPPIE database.[a]

Binding partner	Acronym	HIPPIE score
**Cytoplasmic protein NCK1**	**NCK1**	**0.99**
**Neural Wiskott-Aldrich syndrome protein**	**WASL**	**0.99**
**Wiskott-Aldrich syndrome protein**	**WAS**	**0.99**
Growth factor receptor-bound protein 2	GRB2	0.94
**Src substrate cortactin**	**CTTN**	**0.86**
Tyrosine-protein kinase HCK	HCK	0.85
Cytoplasmic protein NCK2	NCK2	0.82
Homer protein homolog 3	HOMER3	0.73
WW domain-containing protein 2	WWP2	0.73
Transcription elongation factor SPT5	SUPT5H	0.72
Amyloid beta A4 precursor protein-binding family B member 1	APBB1	0.68
**Hematopoietic lineage cell-specific protein**	**HCLS1**	**0.68**
Pre-mRNA-processing factor 40 homolog A	PRPF40A	0.68
WW domain-binding protein 4	WBP4	0.68
Abl interactor 2	ABI2	0.63
BAG family molecular chaperone regulator 3	BAG3	0.63
ELAV-like protein 1	ELAVL1	0.63
Fas-activated serine/threonine kinase	FASTK	0.63
Formin binding protein 2/SLIT-ROBO ρ-GTPase-activating protein 2	SRGAP2	0.63
Tyrosine-protein kinase Fyn	FYN	0.63
Gigaxonin	GAN	0.63
Hematopoietic protein 1	ALAS1	0.63
**Intersectin-2**	**ITSN2**	**0.63**
Leucine-rich repeat serine/threonine-protein kinase 2	LRRK2	0.63
Myc proto-oncogene protein	MYC	0.63
Protein kinase C and casein kinase substrate in neurons protein 1	PACSIN1	0.63
Protein kinase C and casein kinase substrate in neurons protein 2	PACSIN2	0.63
Protein kinase C and casein kinase substrate in neurons protein 3	PACSIN3	0.63
Proline-serine-threonine phosphatase-interacting protein 1	PSTPIP1	0.63
Roundabout homolog 4	ROBO4	0.63
Protein transport protein Sec24C	SEC24C	0.63
SH3 domain-containing kinase-binding protein 1	SH3KBP1	0.63
E3 ubiquitin-protein ligase SH3RF1	SH3RF1	0.63
Actin, alpha cardiac muscle 1	ACTC1	0.62
**Actin, cytoplasmic 2**	**ACTG1**	**0.56**
Cell division control protein 42 homolog	CDC42	0.56
**Crk-like protein**	**CRKL**	**0.55**
Protein kinase C theta type	PRKCQ	0.52
Tyrosine-protein kinase ZAP-70	ZAP70	0.52

[a] Proteins in bold are WIP binding partners mentioned in this review in their structural context
